# Agent-based modeling insights into the optimal distribution of the Fresh Fruit and Vegetable Program

**DOI:** 10.1016/j.pmedr.2020.101173

**Published:** 2020-08-09

**Authors:** Stephanie Schauder, Michael R. Thomsen, Rodolfo M. Nayga Jr

**Affiliations:** a Department of Economics, Cornell University, Uris 429, Ithaca, NY 14853, United States; b Department of Agricultural Economics and Agribusiness, University of Arkansas Division of Agriculture, Agriculture Building 226, Fayetteville, AR 72701, United States; c Food Policy Economics, Department of Agricultural Economics & Agribusiness, University of Arkansas Division of Agriculture, Agriculture Building 217, Fayetteville, AR 72701, United States

**Keywords:** Food Preferences, Fruit, Vegetables, Computer Simulation, Government Programs, Resource Allocation, Pediatric Obesity, Appetite Regulation

## Abstract

The Fresh Fruit and Vegetable Program (FFVP) program provides a free fresh fruit or vegetable snack to children in low-income schools between two and five times a week. This is an important nationwide program, but current funding levels are insufficient to reach all eligible schools. Thus, there is a need to develop insight on contextual factors that may impact the effectiveness of FFVP in facilitating the development of childhood food preferences. An agent-based model of preference formation is used to understand how exposure to FFVP in early elementary school may affect preferences for healthy food by 6th grade. Preferences are modeled using the temporal difference learning algorithm used by Hammond et al. (2012). This model simulates habit formation in the context of food by modeling preference formation in terms of positive or negative feedback about the food most recently consumed. The model is designed to provide insights into how different patterns of exposure to FFVP affect preferences, and how living in a food desert changes the effect of FFVP. We use data on 35,981 students from Arkansas elementary schools (from 2008 to 2016) and information about the commercial food environment in Arkansas communities to populate the model. We find that early FFVP exposure is more beneficial than late exposure conditional on the number of years exposed. We also find that FFVP is more beneficial for children who grow up in environments lacking healthy food.

## Highlights

• Agent-based modeling assists in disseminating FFVP in the most cost-effective manner.
• Early childhood exposure to fruits and vegetables is more effective in influencing preferences than later exposure.
• More consistent exposure to a fruit and vegetable intervention is more effective than sporadic exposure.
• Children living in food deserts may benefit most from FFVP.

## 1 Introduction

Fruits and vegetables have been shown to reduce the risk of a number of health conditions including cancer, heart disease, stroke, cataracts, and lung disease (Dittus et al., 1995, Van Duyn and Pivonka, 2000, Fulton et al., 2016, Godrich et al., 2018). Fruits and vegetables contain relatively more fiber and water than processed foods, and research shows that diets with low calorie density improve weight management (Stelmach-Mardas et al., 2016). On average, children over four years old do not meet the recommended consumption for fruits or vegetables (U S Department of Agriculture, 2010). Inadequate consumption of fruits and vegetables is especially of concern among lower-income children (Rasmussen et al., 2006). As a means of addressing this public health problem, many researchers and policy makers have become interested in dietary interventions to increase fruit and vegetable intake. One such intervention is the U.S. Department of Agriculture’s (USDA) Fresh Fruit and Vegetable Program (FFVP) (US Department of Agriculture Food and Nutrition Service, 2010). FFVP is a federally funded program that provides a free fresh fruit or vegetable snack at least twice a week to children in qualifying schools. The goals of the program are threefold (US Department of Agriculture Food and Nutrition Service, 2010). First, to expand variety of fruits and vegetables experienced; second, to increase fruit and vegetable consumption; and third to impact health by improving diets. In sum, the overall aim of the program is to increase the quality of children’s diets by providing fruit and vegetable snacks, and increasing awareness about healthy eating (Bartlett et al., 2013). Key elements of the program are summarized as follows. First, the program targets high-need elementary schools. FFVP grants are awarded to applicant schools with the highest percentage of students certified for free and reduced-price school-meal benefits.^1^ The snacks are commonly served in the classroom as part of a nutrition education lesson or in an effort to integrate nutrition education into other subject matter lessons.

Habit formation in fruit and vegetable consumption has been widely studied. FFVP has several key elements that have the potential to make it a successful intervention based on this literature. One of the most commonly cited and tested techniques for increasing fruit and vegetable consumption is repeated exposure (Appleton et al., 2018). A number of studies show that exposing infants to fruits and vegetables during weaning is critical for developing acceptance of fruits and vegetables (Spill et al., 2019, Barends et al., 2014, Barends et al., 2013, Maier et al., 2008). However, it is not just infants who benefit, and research shows that even if exposure to fruits and vegetables occurs later in childhood, it can still have a beneficial effect on preferences (Anzman-Frasca et al., 2018). In fact, many studies show that repeated exposure to fruits and vegetables increases childhood fruit and vegetable consumption (Appleton et al., 2018, Nekitsing et al., 2018, Holley et al., 2017). One of the other potentially beneficial elements of FFVP is that teachers are able to participate in the program along with the class (Bartlett et al., 2013). Role models outside the family who encourage fruit and vegetable consumption may help increase fruit and vegetable intake (Godrich et al., 2018). Finally, FFVP is a school-based intervention. The school has been shown to be a beneficial venue for fruit and vegetable interventions because no other institution has as much contact with children on a daily basis (Gaines and Turner, 2009, Evans et al., 2012).

Indeed, research on FFVP specifically shows that the intervention could be promising. FFVP has been shown to increase fruit and vegetable consumption both in and outside of school without changing total calories consumed (Bartlett et al., 2013). This suggests that children in FFVP schools may be consuming more nutrient-dense foods overall. In fact, research shows that students receiving the FFVP intervention consumed fewer soft drinks and candy (Nagata et al., 2012). Olsho et al. (2015) use a regression discontinuity design to evaluate the effects FFVP on children’s fruit and vegetable intake. They find that children in FFVP schools consumed 1/3 cup more fruits and vegetables on average than those who do not attend FFVP schools. The quantity of fresh fruits and vegetables consumed outside of school also increased in students of FFVP schools. Furthermore, a study by Qian et al. (2016) uses a matching technique and difference in differences methodology to evaluate FFVP’s effect on children’s body mass index (BMI). They find significant evidence that FFVP reduces BMI, although the magnitude of the effects is relatively small. Lin and Fly (2016) report that half of children receiving FFVP attribute higher fruit or vegetable consumption to this intervention. Research shows that FFVP increases fruit and vegetable consumption in rural areas more than in urban areas (Lin and Fly, 2016). This could because rural residents live on average further away from grocery stores than urban residents (Dean and Sharkey, 2011). For this reason, we decided to include measures of the food environment in our model.

The ability of economic agents to change and update preferences over time is becoming an increasingly important feature of modern economic choice models (Grüne-Yanoff et al., 2009). The behavioral economics literature finds that past experiences can have an effect on current preferences. Maltz (2016)) presents a model of preference formation where the utility function is updated based on consumption in the previous period. This framework is broad and can accommodate behavioral assumptions such as brand loyalty, status quo bias, variety seeking, etc. In a similar vein, FFVP has been shown to speed the development of preferences for fruits and vegetables (Masis et al., 2017).^2^ Thus, in the model presented below, preferences can be updated and learned based on past exposure.

The dynamics of preference formation used in the model are adapted from the temporal difference learning (TDL) algorithm used in Hammond et al. (2012). The TDL algorithm was created to model the ways that animals make predictions about their future environment in the presence of rewards (Schultz et al., 1997). The TDL algorithm, in contrast to other prediction-learning algorithms, updates preferences based on the error between perceived reward in the current period and next period, rather than the difference between the current period and the final period. The TDL method has been shown to converge faster and produce more accurate predictions than other learning algorithms (Sutton, 1988). The processes of updating preferences with exposure approximates dopamine signals as it pertains to food (Hammond et al., 2012). Hammond et al. (2012) apply the TDL algorithm within an agent-based model to better understand the context of habit formation for healthy and unhealthy food. Their model shows a “lock in effect” with respect to food choices where the initial food environment has a strong influence on later food choices.

To the extent that the FFVP facilitates formation of preferences for target foods, it is important to understand how preference learning depends on the age at which children are first exposed to the program and duration of exposure. It is also important to develop insights into the role of environmental context on program effectiveness. Questions relating to timing, duration, and context are relevant because FFVP funding does not cover all eligible elementary schools. Thus, it is possible for a school to receive an FFVP grant in one year but not in another due to fluctuations in free and reduced meal eligibility among its student body and those of other applicant schools.

This study uses an agent-based model of preference learning to better understand the effect of timing and duration of FFVP exposure during elementary school on the development of preferences for healthy foods like fruits and vegetables. The model is calibrated to earlier findings on number of servings after one year of FFVP exposure (Bartlett et al., 2013, Olsho et al., 2015). The state of Arkansas provides the context for the model. Arkansas is an interesting and important case to study since it has one of the highest childhood obesity rates in the US (i.e., the 7th most obese state, with an obesity rate of 35%) (National Cancer Institute, 2018). Furthermore, in Arkansas 50.9% of adolescents report consuming fruit less than 1 time daily and 48.3% of students report consuming vegetables less than 1 time daily (Centers for Disease Control and Prevention, 2019). These statistics suggest it is one of the states where the FFVP program could potentially make the greatest impact. There are many obstacles to consuming fruits and vegetables. These include affordability, lack of time, or lack of access to healthy food (Dave et al., 2017). Therefore, it is important to understand whether increasing exposure to fruits and vegetables in states like Arkansas can help improve nutrition.

Given this context, patterns of FFVP exposure used in the model conform to actual exposure patterns observed in Arkansas public schools from the inception of FFVP in the 2008/2009 academic year through the 2015/2016 academic year. The model also reflects the commercial food environment facing Arkansas public elementary schoolchildren that were eligible for FFVP. While we do not have direct evidence of the impact of the food environment on FFVP effectiveness, we choose to explore this element because Lin and Fly (2016) suggest that FFVP may impact students differentially depending on their environment. One advantage of a modeling exercise is to understand the potential importance of the environment on FFVP effectiveness over time, which can then be tested in future empirical studies.

Agents in the model conform to the 2008/2009 and 2009/2010 kindergarten cohorts. These agents are followed through sixth grade in 2014/2015 and 2015/2016, respectively. Thus, the model can be used to explore potential differences in the degree of preference learning among FFVP exposed and non-exposed children by age 12. We know that children in FFVP schools consume more fruits and vegetables on average (Bartlett et al., 2013). The objective of this model is to apply the literature on preference formation to the context of FFVP to understand how changing preferences may improve consumption of fruits and vegetables.

## 2 Methods

The agent-based model incorporates the TDL algorithm to assess learning of preferences for a healthy food (e.g., fruits and vegetables). Following Hammond et al. (2012) preference dynamics are presented in terms of the agent’s true valuation of food group *j* ($V_{\mathrm{ij}}$) and his or her perceived valuation:

(1) $${\hat{V}}_{\mathrm{ij}}\left(t+1\right)={\hat{V}}_{\mathrm{ij}}\left(t\right)+\alpha_{i}\left[V_{\mathrm{ij}}-{\hat{V}}_{\mathrm{ij}}\left(t\right)\right]$$

where $\alpha_{i}$ is the speed at which the agent *i* learns, $V_{\mathrm{ij}}$ is agent i’s true valuation of food *j* and ${\hat{V}}_{\mathrm{ij}}\left(t\right)$ is the agent’s perceived valuation at time t. In our implementation of the model, ${0<\hat{V}}_{\mathrm{ij}}\left(t\right)\leq V_{\mathrm{ij}}$. When preferences are fully learned, ${\hat{V}}_{\mathrm{ij}}\left(t\right)=V_{\mathrm{ij}}$. The true valuation is further specified as

(2) $$V_{\mathrm{ij}}=\beta_{j}$$

where $\beta_{j}$ is the “intrinsic palatability” of food *j* (the same for all agents). Our model includes two types of food: healthy food (H) and unhealthy food (U), so *j* ∈ (H, U). We are primarily interested in the formation of preferences for healthy food and assume that preferences for unhealthy food are fully formed at the starting point of the model (${\hat{V}}_{\mathrm{iU}}\left(0\right)=V_{\mathrm{iU}}=1$ for all *i*).^3^ In contrast, preferences for healthy food at the start of the model are completely unformed (${\hat{V}}_{\mathrm{iH}}\left(0\right)=0)$ and the true value of healthy food is normalized to 1 ($V_{\mathrm{iH}}=1$)^4^

As in Hammond et al. (2012), preference learning in our model depends on the food environment confronting the agent. At each step of the model simulation, the agent makes a food choice in one of two situations. In the first situation, the agent is only able to consume the healthy food (the healthy situation). In the second situation, the agent has a choice between healthy and unhealthy food (the choice situation). The probability of the agent landing in the healthy situation is γ. The parameter γ is determined by whether the agent is receiving FFVP and whether the agent lives in an unhealthy or healthy food environment (e.g., food desert or non-desert). If the agent lives in an unhealthy food environment, the probability of landing in a healthy situation is a uniform random draw between (0, $p_{1}$). If the agent is in a healthy food environment, the probability of landing in a healthy situation is a uniform random draw between ($p_{1}$, $p_{2}$), with $p_{2}>p_{1}$. Regardless of the agent’s food environment, if the agent receives FFVP in a given year the uniform random draw is augmented by a constant *a*. The parameters $p_{1}$,$p_{2}$, and *a* are then chosen in the calibration below. The following equation summarizes these statements:

(3) $$\gamma \in \left\{\begin{array}{cc} {(0,p}_{1}) & \mathrm{if}\;an\;unhealthy\;environment\;and\;no\;FFVP\;exposure\\ {(0,ap}_{1}) & \mathrm{if}\;an\;unhealthy\;environment\;with\;FFVP\;exposure\\ {(p_{1},p}_{2}) & \mathrm{if}\;a\;healthy\;environment\;and\;no\;FFVP\;exposure\\ {({\mathrm{ap}}_{1},ap}_{2}) & \mathrm{if}\;a\;healthy\;environment\;with\;FFVP\;exposure \end{array}\right)$$

When the agent consumes healthy food (either in the healthy situation or the choice situation), ${\hat{V}}_{\mathrm{iH}}\left(t\right)$, perceived preferences for healthy food, is updated according to equation (1). When the agent is in the choice situation, the agent chooses the healthy food with probability $P_{\mathrm{iH}}\left(t\right)$ defined by:

(4) $$P_{\mathrm{iH}}\left(t\right)=\frac{{\hat{V}}_{\mathrm{iH}}\left(t\right)}{{\hat{V}}_{\mathrm{iU}}\left(t\right)+{\hat{V}}_{\mathrm{iH}}\left(t\right)}$$

where ${\hat{V}}_{\mathrm{iU}}\left(t\right)$ is the agent specific preference for unhealthy food. As noted above, preferences for unhealthy food are assumed to be fully formed. Therefore, ${\hat{V}}_{\mathrm{iU}}\left(t\right)=1$ for all agents.^5^ Thus, if unhealthy food is chosen, preferences for healthy food do not change. As agents discover their preferences for the healthy food, the likelihood that the healthy food will be chosen in choice environments increases and this can accelerate learning.

Because preferences are not directly observed and given the need to ground the preference learning model in reality, there is a need to convert preferences into choice outcomes. To do this, we require that ${0\leq \hat{V}}_{\mathrm{iH}}\left(t\right)<1$. The number of servings of the healthy food (e.g., fruits and vegetables) chosen by the agent is a random draw from the inverse Poisson density function, $f\left({\hat{V}}_{\mathrm{iH}}\left(t\right),\lambda \right),$ with mean of λ. This assumption allows the model to be calibrated to earlier findings on the impact of FFVP on servings of fruits and vegetables (Olsho et al., 2015). The Poisson distribution represents the variation in preferences among students. While the mean servings might be λ; some students will consume more or less depending on their specific like or dislike of healthy food.^6^ The model was calibrated to Olsho et al. (2015), please see Appendix 1 for more details.

### 2.1 Model implementation

The model was simulated over 1260 iterations. This corresponds to 180 school days per year and 7 academic years (kindergarten through sixth grade). The model is used to assess the impact of the program on Arkansas children’s preference learning and intake of fruits and vegetables by age 12 (grade 6).^7^ The model simulations track the 2008/2009 and 2009/2010 kindergarten cohorts (35,981 studentagents) through sixth grade using data from the 2008/2009 to 2015/2016 academic years from the Arkansas Department of Education Child Nutrition Unit (Please see complete description of data in Appendix 2).

To model the Arkansas context, we collected data from National Center for Educational Statistics Common Core of Data file, the American Community Survey 5-year Summary files, Arkansas Department of Education Child Nutrition Unit, and from the ReferenceUSA® database. See Appendix 2 for specific details about these data sources.

Because schools may receive FFVP in some years but not others, we analyze the effect of different patterns of student exposure to FFVP on preference learning. For the rest of this paper, we refer to the exposure pattern with a 7-digit binary identifier with digits corresponding to the grade in school (K, 1, 2, 3, 4, 5, and 6). In this identifier, 1 and 0 indicate that the agent attended or did not attend an FFVP school in a given grade, respectively. For example, an indicator of 1010000, indicates FFVP exposure during kindergarten (first digit of the indicator) and grade 2 (third digit of the indicator)

## 3 Results

Table 1 presents summary statistics for all agents in the simulation. We followed the 278 Arkansas elementary schools over the study period that were eligible for, but that may or may not have received FFVP in any given year (a school can switch from not receiving FFVP to receiving FFVP and vice versa). Similarly, a census block could change desert status over time.^8^ As shown in Table 1, 70.7% of students lived in a food desert at some point over the period of the analysis (i.e., 7 years). Preferences for healthy food, $V_{\mathrm{iH}}$, are almost fully formed by 6th grade,^9^ and 45% of agents were exposed to FFVP at least once.

**Table 1:** Summary Statistics from the Model Results for Simulated Arkansas Students: Individual Level (N = 35,981).

Variable	Mean	Std. Dev.	Min	Max
*V_iH_* by 6th grade	0.889	0.070	0.142	0.982
*P_iH_* by 6th grade	0.469	0.022	0.124	0.496
Ever Exposed to FFVP	0.290	0.454	0.000	1.000
Ever Lived in a Food Desert	0.707	0.455	0.000	1.000

Notes: Individuals are simulated students from Arkansas schools (2008–2016). *V_iH_* is agent *i*'s preference for healthy food *H* and ranges between 0 and 1. *P_iH_* is the probability that agent *i* chooses healthy food *H* when placed in a choice environment.

In our data, only 104 agents, received FFVP in each grade (1111111), while 25,540 agents never received FFVP (0000000). The remaining 10,337 agents received FFVP in some but not all grades. There are a total of 128 ($2^{7}$) possible FFVP-exposure combinations but we only observe 40 of these in our simulation dataset based on Arkansas FFVP awards to schools during the study period that was modeled.

**Fig. 1:**
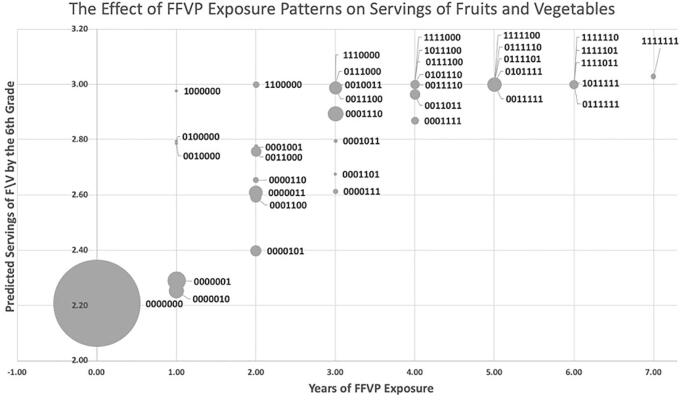
The Effect FFVP Exposure Patterns on Predicted Fruit and Vegetable Consumption. In the 7-digit binary exposure patterns, values of 0 and 1 indicate years the child attended a school with and without FFVP, respectively (ex: 0001100 means the child was exposed to FFVP only in 3rd and 4th grade). The bubble size corresponds to the number of students in each category. Individuals are simulated students from Arkansas schools (2008–2016).

Fig. 1 shows all 40 exposure patterns and how each pattern is associated with predicted servings of fruits and vegetables in the 6th grade. The bubble size corresponds to the number of agents in each exposure pattern. The positive association between servings and years of exposure indicates that multiple years of FFVP exposure is beneficial. Another takeaway from data summarized in Fig. 1 is that receiving FFVP early in an agents’ elementary school experience has a greater effect on servings consumed in 6th grade than receiving FFVP in later years. In fact, the model predicts that continuous exposure in grades K-2 (1110000) is as effective as continuous exposure of grades K-6 (1111111). This makes sense because in the model, preferences are learned but are not forgotten. Consequently, early exposure to FFVP facilitates preference learning and increases the likelihood that the healthy food will be selected when the agent confronts a choice in the future thereby facilitating complete learning by grade 6.

**Fig. 2:**
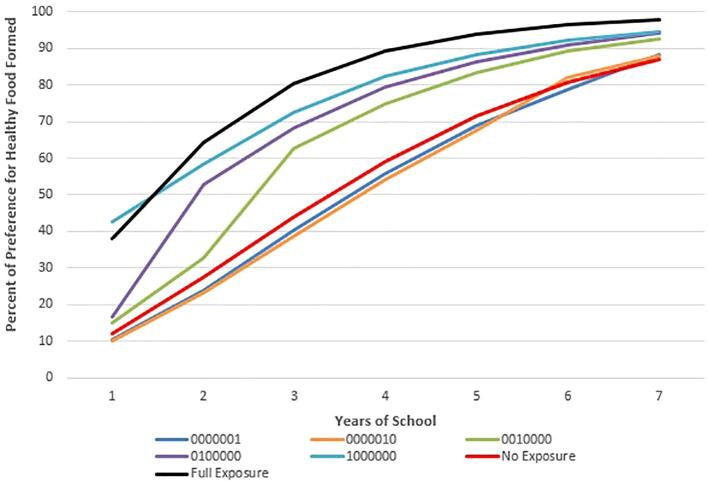
The Effect of One Year of FFVP Exposure on Preferences for Healthy Food by Exposure Pattern Compared to the Fully-Exposed and Never-Exposed Reference Patterns. In the 7-digit binary exposure patterns, values of 0 and 1 indicates years the child attended a school with and without FFVP, respectively (ex: 0001100 means the child was exposed to FFVP only in 3rd and 4th grade). Individuals are simulated students from Arkansas schools (2008–2016).

**Fig. 3:**
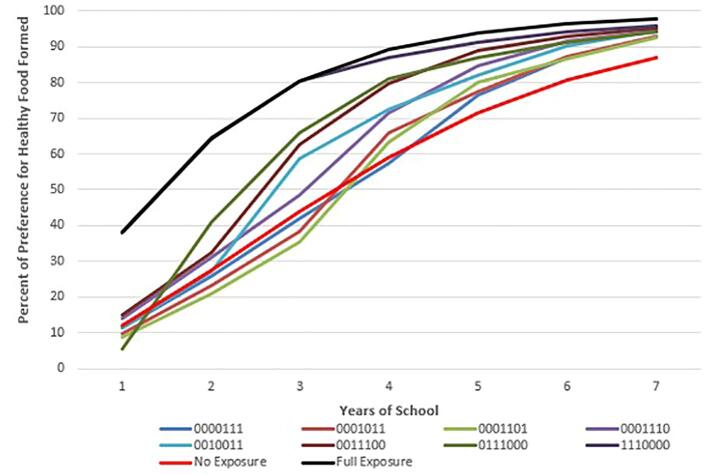
The Effect of Three Years of FFVP Exposure on Preferences for Healthy Food by Exposure Pattern Compared to the Fully-Exposed and Never-Exposed Reference Patterns. In the 7-digit binary exposure patterns, values of 0 and 1 indicates years the child attended a school with and without FFVP, respectively (ex: 0001100 means the child was exposed to FFVP only in 3rd and 4th grade). Individuals are simulated students from Arkansas schools (2008–2016).

**Fig. 4:**
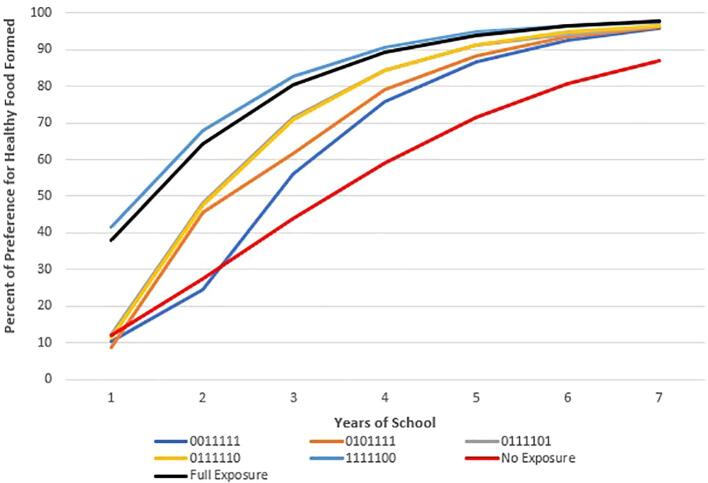
The Effect of Five Years of FFVP Exposure on Preferences for Healthy Food by Exposure Pattern Compared to the Fully-Exposed and Never-Exposed Reference Patterns. In the 7-digit binary exposure patterns, values of 0 and 1 indicate years the child attended a school with and without FFVP, respectively (ex: 0001100 means the child was exposed to FFVP only in 3rd and 4th grade). Individuals are simulated students from Arkansas schools (2008–2016).

Fig. 2, Fig. 3, Fig. 4 show examples of the evolution of preferences over time for agents who were exposed to FFVP for 1, 3, and 5 years respectively. We also include in these graphs (for reference) the trajectory of preference learning for those who had no exposure (0000000) and full exposure (1111111). Interestingly, no exposure has almost an identical effect on preferences as one year of exposure in the fifth or sixth grade (Fig. 2), whereas exposure in kindergarten or first grade improves preferences immensely. These preference learning patterns are consistent with data presented in Fig. 1. Fig. 3, Fig. 4 show a similar pattern except that because the exposure was over a longer number of years, all agents have much healthier preferences than their counterparts who do not receive FFVP.

**Fig. 5:**
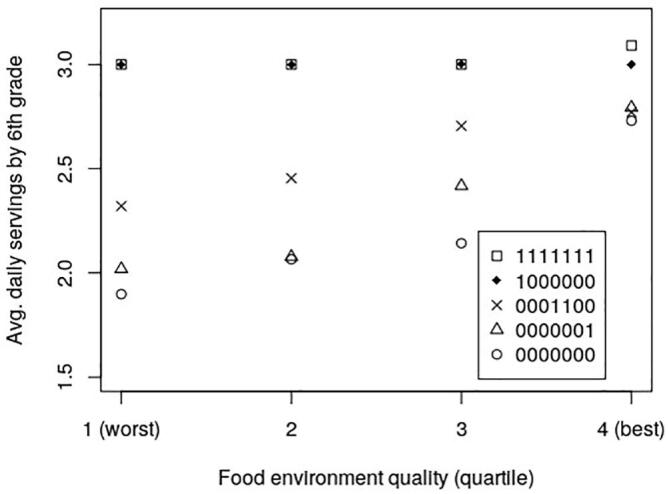
The Effect of the Food Environment and Exposure to FFVP on Servings of Fruits and Vegetables. In the 7-digit binary exposure patterns, values of 0 and 1 indicate years the child attended a school with and without FFVP, respectively (ex: 0001100 means the child was exposed to FFVP only in 3rd and 4th grade). Individuals are simulated students from Arkansas schools (2008–2016).

**Fig. 6:**
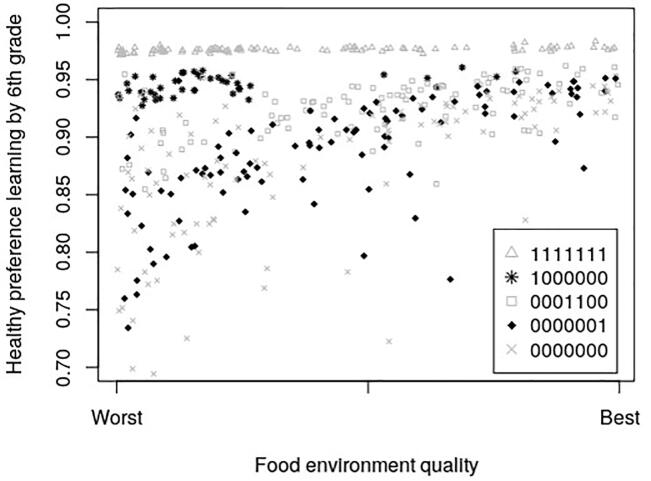
The Effect of the Food Environment and Exposure to FFVP on Preference Formation for Healthy Food. In the 7-digit binary exposure patterns, values of 0 and 1 indicate years the child attended a school with and without FFVP, respectively (ex: 0001100 means the child was exposed to FFVP only in 3rd and 4th grade). Individuals are simulated students from Arkansas schools (2008–2016).

Fig. 5, Fig. 6 show how the food environment impacts the preferences and consumption of students of different selected exposure patterns. For students exposed to FFVP every year, food desert status matters very little. However, for those with much less exposure to FFVP, food deserts are detrimental to healthy preference formation.

Table 2 presents the disparate effect of food environment on servings of fruits and vegetables consumed by FFVP exposure. For agents who never resided in a food desert, receiving FFVP causes an increase in fruit and vegetable consumption of 0.43 servings, while for those in a food desert, receiving FFVP causes an increase in consumption of 0.64 servings. Both of these differences from the model are highly significant.

**Table 2:** Mean Predicted Servings of Fruits and Vegetables by the 6th Grade for Simulated Arkansas Students.

	Never Received FFVP		Received FFVP^a^		Difference
Food Environment	N	Mean (Std. Dev.)	N	Mean (Std. Dev.)	t-statistic^b^ (p-value)
Never in a Food Desert	7942	2.493	2591	2.924	−55.928
		(0.500)		(0.269)	(0.000)
In a Food Desert^c^	17,598	2.079	7850	2.716	−100.97
		(0.472)		(0.461)	(0.000)
Total	25,540	2.208	10,441	2.767	−105.200
		(0.518)		(0.431)	(0.000)

Notes: ^a^Received FFVP at least once between kindergarten and sixth grade. ^b^The t-statistic is for the null hypothesis of equal means and is computed under the assumptions of independence allowing for unequal variances between the exposed and never-exposed samples. **^c^**In a food desert at least once between kindergarten and sixth grade. Individuals are simulated students from Arkansas schools (2008–2016).

## 4 Discussion

Using an adaptation of the Hammond et al. (2012) model of preference formation, we examined the effect of FFVP participation on elementary school children’s fruit and vegetable consumption to better understand how the timing and duration of a fruit and vegetable intervention can affect healthy eating. Our results yield two main testable hypotheses that should be examined with future empirical work. First, early exposure to FFVP may be more beneficial than late exposure to the formation of preferences by the 6th grade. Early consistent exposure is the most effective intervention. Second, we find that FFVP may be more beneficial for those children living in food deserts than for children living in non-food deserts. In fact, the model suggests is that an intervention like FFVP may be able to offset the disadvantages of a poor food environment and thereby help address disparities in diet and health.

An important implication of the results is that early exposure to food stimuli has a much more potent effect on eating behavior than later exposure, consistent with the results from Hammond et al. (2012). Additionally, other research shows that FFVP has the ability to alter preference formation. FFVP increased the likelihood that children asked for fruits and vegetables while shopping and that they chose fruits and vegetables at home (Ohri-Vachaspati et al., 2018). As compared to students at control schools, students receiving FFVP displayed increased willingness to try new fruits and vegetables (Jamelske et al., 2008).

Although our model is calibrated to Olsho et al. (2015), a limitation of our paper is that we do not have long term data on the effects of FFVP. Our model is currently best understood as an application of the Hammond et al. model (2012). Thus, the strength of this study is not in what it says about the magnitude of the effect of FFVP, but rather in what it says about how the effect varies by exposure pattern and food access.

Another limitation of our paper is that the agent-based model yields results from simulated preferences, but we cannot verify if this is consistent with actual preference formation among children receiving FFVP. However, this model is important because it yields testable hypotheses related to duration and age of exposure that if verified empirically could be used to help optimally distribute FFVP funding. For example, based on our results, it is possible that providing FFVP to more students, for fewer years, but focusing on the earliest years of elementary school would have equal or greater aggregate impact. Additionally, given the large effects we observed in children with limited food access, it might be cost effective to concentrate on the children who live in food desert areas.

## 5 Conclusion

This paper models preference formation for healthy food among children receiving FFVP. It reveals several important testable hypotheses, namely that early exposure to FFVP is more beneficial than late exposure, and that the FFVP intervention is most effective for those children living in food deserts. In future work we plan to collect data which will allow us to test these hypotheses empirically. This will help policy makers understand how to optimally distribute government funds so as to maximize impact.

Our study is primarily based in Arkansas. FFVP is national in scope, but we only have data on Arkansas FFVP awards and therefore calibrated our model to an Arkansas context. This could impact generalizability because FFVP grants do not cover all eligible schools and neediest schools get priority. In states with a more equitable distributions of income or less income segregation, FFVP awards could go to schools with lower proportions of free/reduced lunch eligibility. As an extension, we would like to collect data from different states to see if results are consistent.

As the FFVP program continues, it would also be very useful to collect data from middle and high school students who were exposed to FFVP during their time at the elementary school and an appropriate control group. This would allow us to understand how long the FFVP intervention persists. With such data, we could further calibrate our model accordingly to better understand preference formation as it pertains to fruits and vegetables, which would help policy makers improve FFVP and design similar interventions.

## Funding/financial disclosures

This research was supported in part by the National Institute of General Medical Sciences of the 10.13039/100000002National Institutes of Health under Award Number P20GM109096. The content is solely the responsibility of the authors and does not necessarily represent the official views of the National Institutes of Health.

## Conflicts of interest

Authors declare no conflicts of interest.

## CRediT authorship contribution statement

**Stephanie Schauder:** Conceptualization, Methodology, Software, Formal analysis, Writing - original draft, Writing - review & editing, Visualization. **Michael R. Thomsen:** Conceptualization, Software, Formal analysis, Data curation, Writing - original draft, Writing - review & editing, Supervision. **Rodolfo M. Nayga Jr:** Conceptualization, Methodology, Data curation, Resources, Writing - review & editing, Supervision.

## Appendix 1 Model calibration

The model was calibrated to Olsho et al. (2015). Their study uses a regression discontinuity design to measure differences in consumption of fruits and vegetables among fouth to sixth graders. To calibrate our model, we simulate the formation of preferences from kindergarten to fourth grade. In the calibration, each agent was assigned a starting value of ${\hat{V}}_{\mathrm{iH}}\left(0\right)=0.01$. For the calibration, our model was simulated over 900 iterations corresponding to 180 school days over five academic years, the idea being to model preference development from kindergarten through the fourth grade. We do not consider food deserts in the calibration so ∈ ${(0,p}_{2})$. For the first years, preferences are modeled as if no students receive FFVP. For the last 180 iterations (the fourth grade year), half of the children do not receive FFVP and the γ remains the same as above (γ∈ ${(0,p}_{2})$). The other half of the students do receive FFVP and γis multiplied by a constant *a*. The values of *a* and $p_{2}$ were chosen to create an average 1/3 cup difference between the agents in the augmented and unaugmented fourth grade food environments. This 1/3 cup difference corresponds to the estimated impact of one-year’s exposure to FFVP reported in the Olsho et al. study (2015). The parameter, $p_{1}$, was chosen so as to preserve the mean γ value. The parameter λ was chosen so that the mean consumption of fruits and vegetables for agents not receiving FFVP matches the value found in the national statistics, 2.2 servings for 9–13 year olds (National Cancer Institute, 2018). These parameters are $p_{H}$ = 1, α=0.005,$\beta_{H}=0.99$, λ = 1, $p_{1}=0.05$, $p_{2}=0.2$ and *a* = 0.4.

## Appendix 2 Data sources

The model was populated with agents reflecting the enrollment data from the Arkansas school system. These data were coupled with historical records of FFVP grants awarded to schools in the state. We also incorporated Census block-level data on the commercial food environment from the communities where these schools drew children.

The location and composition of schools was obtained from the National Center for Education Statistics Common Core of Data files (National Center for Education Statistics, 2020). Block-group level income and poverty measures were obtained from the American Community Survey 5-year Summary files (U.S. Census Bureau, 2020). A list of schools participating in FFVP by year (2008/2009–2015/2016) were obtained from the Arkansas Department of Education Child Nutrition Unit (Arkansas Department of Education, 2020).

To measure the quality of the food environment, we identified supermarkets, and supercenters from the ReferenceUSA® database (ReferenceUSA, 2020). This database provides validated store locations as they existed in each year of our study. Supermarkets and supercenters were identified from the establishments in the ReferenceUSA® database by an inspection of trade name, standard industrial classification code, North American Industry Classification Code, and through internet searches on store name and location. We then assigned each of the more than 186,000 Census blocks in Arkansas as urban or rural using Census-defined places and then computed the number of supermarkets or supercenters within one mile of urban blocks and 10 miles of rural blocks. Food desert classification depends on (a) the absence of supermarkets/supercenters within the one and ten-mile radii for urban and rural blocks, respectively; and (b) the income status of the block-group. Low-income block groups are defined as those with more than 20 percent of the population below poverty or with median household income below 80 percent of the statewide median income. Food-desert blocks are both low-income and low access (Ver Ploeg and Rhone, 2017).

In the calibration exercise described above, the likelihood of the agent encountering a healthy situation (denoted by γ) is a uniform draw over the interval ${(0,p}_{2})$. However, in the simulations, this likelihood is also allowed to depend on the agent’s food desert status in any given year. This is accomplished by assigning each agent lower and higher likelihoods of encountering a healthy situation from the interval ${(0,p}_{1})$ and ${(p_{1},p}_{2})$, respectively, as indicated in equation (3). The lower likelihood is used in years where the agent’s census block was classified as a food desert while the higher likelihood is used during years when the agent’s census block was a non-desert.

We limit our dataset to public elementary schools (grades kindergarten to 6th grade) with at least 50% of students receiving free or reduced lunch because these are the only schools eligible to receive the FFVP intervention. Selection is based on the number of students receiving free and reduced lunch and the school’s plan to implement FFVP (Bartlett et al., 2013). Therefore, our comparison group is schools that were eligible, but did not receive FFVP.

## Footnotes

1 States are required to conduct outreach to the highest need schools to ensure that these schools apply and meet the criteria to participate in the program. Second, once an FFVP grant has been awarded, snacks are provided to all children attending the school, regardless of the child’s own family income. Finally, FFVP snacks are required to be served during normal school hours but outside of the school meal times.

2 The development of preferences depends on exposure. When children are not exposed to a new food, they will not have a preference for or against it. Increasing exposure increases the rate at which children learn which foods they enjoy.

3 .Even though preferences for unhealthy foods are fully learned during the initial period, the likelihood of choosing unhealthy foods declines as agents develop preferences for healthy foods during the model steps. Whether programs like FFVP actually reduce the intrinsic palatability of unhealthy foods is an interesting issue but we do not model it here to maintain model tractability and because of limited data and earlier findings on this issue.

4 Because preferences for unhealthy food are assumed to be fully formed in this model. The V_ij_(t) of healthy food is normalized to 1. In short, there are likely to be differences between dopamine signaling between healthy food and unhealthy food, just as there are likely differences between fruits and vegetables, just as there could be differences in dopamine signaling within the categories of fruits and vegetables. However, for the purposes of this model we are only looking at the formation of preferences for one type of food, so relative differences do not matter. However, as an extension, research could look at the simultaneous formation of preferences for healthy and unhealthy food, where the relative palatability would be important.

5 Hammond et al. explore preference learning for healthy and unhealthy food simultaneously, and this is something that could be incorporated in future versions of the model Hammond et al. (2012).

6 In the original Hammond et al. (Hammond et al., 2012)model V_ij_(t) can be further broken down into V_ij_(t)=B_ij_p_j_ where p_j_ is the baseline palatability of food j, and B_ij_ is individual i’s proclivity for food j. In our model, we have V_ij_(t)=B_j_ where B_j_ is the intrinsic palatability of food j. We do not include individual specific variation in preferences because that is built into the model by making the number of servings of fruit and vegetables consumed be a random draw from the Poisson distribution.

7 FFVP is an elementary school intervention and very few schools that qualify for the program house children beyond grade 6.

8 One of the key ways that blocks can change desert status is if the average income in the census block changes. Another way is if grocery stores enter or go out of business. See Rhone (2010) for statistics about how food deserts change at the national level.

9 When preferences are fully formed $V_{\mathrm{iH}}=1$.
